# A Low-Dose β1-Blocker in Combination with Milrinone Improves Intracellular Ca^2+^ Handling in Failing Cardiomyocytes by Inhibition of Milrinone-Induced Diastolic Ca^2+^ Leakage from the Sarcoplasmic Reticulum

**DOI:** 10.1371/journal.pone.0114314

**Published:** 2015-01-23

**Authors:** Shigeki Kobayashi, Takehisa Susa, Hironori Ishiguchi, Takeki Myoren, Wakako Murakami, Takayoshi Kato, Masakazu Fukuda, Akihiro Hino, Takeshi Suetomi, Makoto Ono, Hitoshi Uchinoumi, Hiroki Tateishi, Mamoru Mochizuki, Tetsuro Oda, Shinichi Okuda, Masahiro Doi, Takeshi Yamamoto, Masafumi Yano

**Affiliations:** Division of Cardiology, Department of Medicine and Clinical Science, Yamaguchi University Graduate School of Medicine, Ube, Japan; Loyola University Chicago, UNITED STATES

## Abstract

**Objectives:**

The purpose of this study was to investigate whether adding a low-dose β1-blocker to milrinone improves cardiac function in failing cardiomyocytes and the underlying cardioprotective mechanism.

**Background:**

The molecular mechanism underlying how the combination of low-dose β1-blocker and milrinone affects intracellular Ca^2+^ handling in heart failure remains unclear.

**Methods:**

We investigated the effect of milrinone plus landiolol on intracellular Ca^2+^ transient (CaT), cell shortening (CS), the frequency of diastolic Ca^2+^ sparks (CaSF), and sarcoplasmic reticulum Ca^2+^ concentration ({Ca^2+^}_SR_) in normal and failing canine cardiomyocytes and used immunoblotting to determine the phosphorylation level of ryanodine receptor (RyR2) and phospholamban (PLB).

**Results:**

In failing cardiomyocytes, CaSF significantly increased, and peak CaT and CS markedly decreased compared with normal myocytes. Administration of milrinone alone slightly increased peak CaT and CS, while CaSF greatly increased with a slight increase in {Ca^2+^}_SR_. Co-administration of β1-blocker landiolol to failing cardiomyocytes at a dose that does not inhibit cardiomyocyte function significantly decreased CaSF with a further increase in {Ca^2+^}_SR_, and peak CaT and CS improved compared with milrinone alone. Landiolol suppressed the hyperphosphorylation of RyR2 (Ser2808) in failing cardiomyocytes but had no effect on levels of phosphorylated PLB (Ser16 and Thr17). Low-dose landiolol significantly inhibited the alternans of CaT and CS under a fixed pacing rate (0.5 Hz) in failing cardiomyocytes.

**Conclusion:**

A low-dose β1-blocker in combination with milrinone improved cardiac function in failing cardiomyocytes, apparently by inhibiting the phosphorylation of RyR2, not PLB, and subsequent diastolic Ca^2+^ leak.

## Introduction

Aberrant Ca^2+^ release through the cardiac ryanodine receptor (RyR2), which represents diastolic Ca^2+^ leak from sarcoplasmic reticulum (SR), is a major cause of heart failure and lethal arrhythmia [1, 2].

In heart failure, diastolic Ca^2+^ leak from SR and decreased Ca^2+^ uptake to SR causes intracellular Ca^2+^ overload as well as depression of SR Ca^2+^ content, eventually leading to systolic and diastolic left ventricular (LV) dysfunction [1, 2]. Moreover, diastolic Ca^2+^ leak from SR via RyR2 can initiate delayed afterdepolarization and trigger activity, leading to arrhythmia [1, 2]. Therefore, RyR2 stabilization may be a novel therapeutic strategy against heart failure and subsequent lethal arrhythmia [1, 2, 3–6].

Short-term inotropic therapy may benefit patients with acute decompensated heart failure (ADHF) corresponding to Forrester subset IV by reducing symptoms and improving endo-organ perfusion [7, 8]. However, it has not demonstrated positive results [9]. Inotropes including dobutamine, dopamine, and phosphodiesterase III inhibitor (i.e., milrinone) have cardiotoxic and arrhythmogenic actions induced by intracellular Ca^2+^ overload [10, 11].

**Figure 1:**
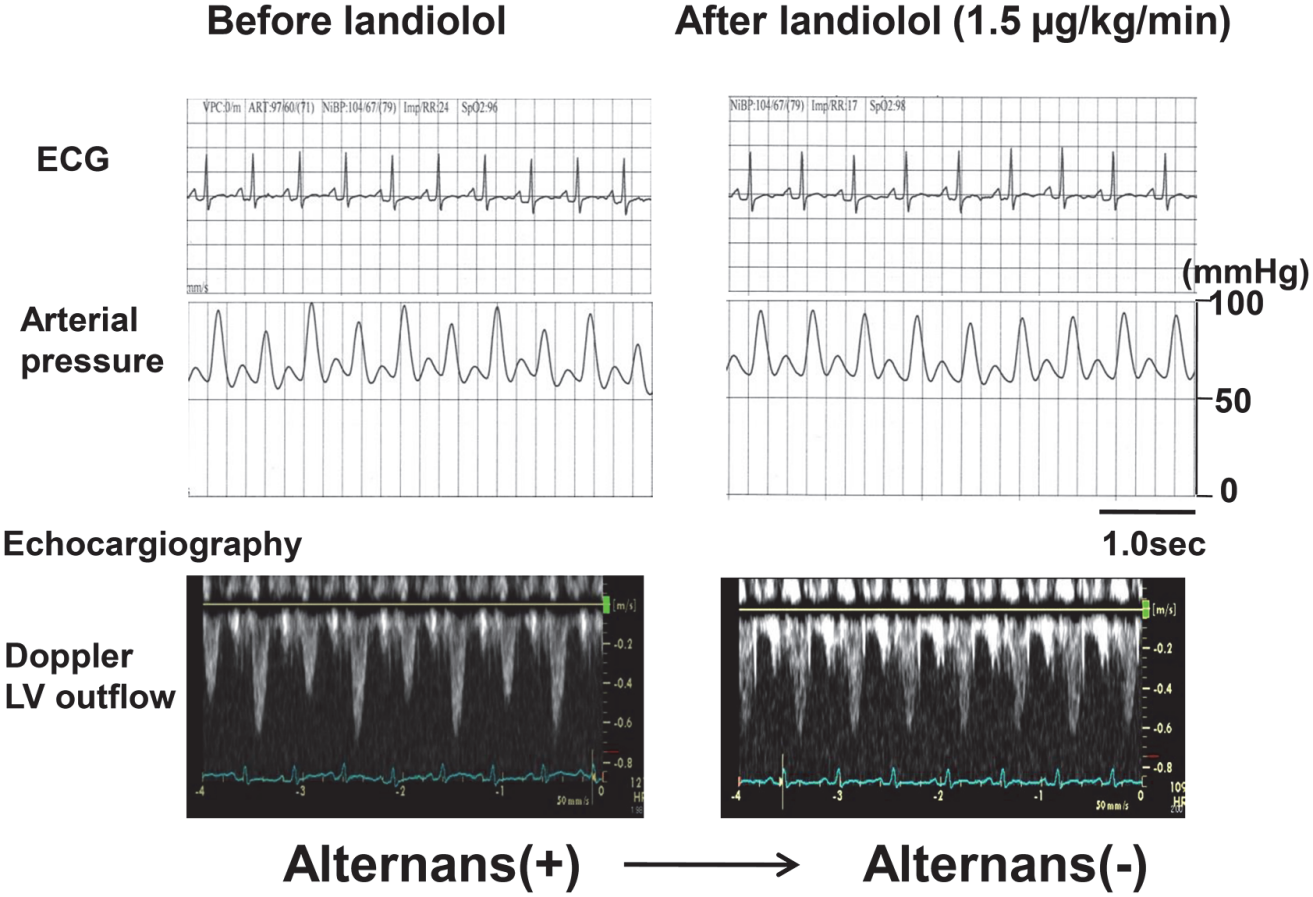
Electrocardiogram, radial arterial pressure, and Doppler left ventricle outflow before and after low-dose landiolol addition to milrinone. Addition of a low-dose β1 blocker (1.5 μg/kg/min) to milrinone eliminated pulsus alternans in a patient with acute decompensated heart failure.

The use of a β-blocker in combination with inotropic agents to treat ADHF has been contraindicated. In cases where acute heart failure with tachycardia is refractory to standard treatments such as diuretics, vasodilators, and milrinone (i.e., heart rate slowing is not observed), a low-dose β-blocker might be effective for treating ADHF, if it has modest negative chronotropic but few cardiosuppressive effects. Landiolol (ONOACT; Ono Pharmaceutical, Osaka, Japan) is the most ultrashort-acting intravenous (elimination t_1/2_: 4 min) and β1-selective adrenergic receptor blocker (β_1_/β_2_ = 255), similar to esmolol, with a significant chronotropic effect and little or no negative inotropic effect at low doses [12–15]. Very recently, this unique β1-blocker was recommended for use in atrial fibrillation and atrial flutter with tachycardia by the Japanese Circulation Society, even for patients with acute heart failure with LV dysfunction [16, 17, 18]. We reported that the addition of low-dose landiolol to milrinone effectively improved cardiac function and eliminated pulsus alternans in 20 patients with ADHF with tachycardia, while standard therapy with diuretics, vasodilators, and milrinone was ineffective in slowing HR [15]. Surprisingly, pulsus alternans disappeared upon addition of low-dose landiolol to milrinone in all affected patients [15]. Before beginning the present study, we reconfirmed the observation that a low dose β1-blocker eliminated alternans of radial arterial pressure and Doppler LV outflow in a patient with severe heart failure, as shown in Fig. 1.

The molecular mechanism underlying how low-dose β1-blocker combined with milrinone affects intracellular Ca^2+^ handling in heart failure remains unclear. One putative mechanism is through slowing HR, which decreases myocardial oxygen demand and improves diastolic filling [15]. From several reports [19–23], moreover, another contributing mechanism might be correction of aberrant intracellular Ca^2+^ handling. In the present study, we investigated the cardioprotective mechanism of a low-dose β1-blocker in intact failing canine cardiomyocytes to clarify the acute effect of low-dose β1-blocker on Ca^2+^ handling at a steady pacing rate of 0.5 Hz. Acute effect of low-dose β1-blocker was defined as add-on effect without long incubation.

## Methods

### Canine heart failure model induced by rapid right ventricular pacing

Dogs (obtained from KITAYAMA LABES CO., LTD, Japan) used in the present study, were all female beagle dogs (10–13 kg in body weight, 3–4 years old). Housing and husbandry conditions at Science Research Center at Yamaguchi University are as follows (see S1 ARRIVE Checklist).

1. Housing: A large separate gage (D90cm x W85cm x H80cm) were given to each dog (number of gage was 12)
2. Husbandry condition: light /dark cycle (12hrs/12hrs) 7am–7pm; temperature 70°F ± 2°F; Food; food for experimental animals (TC-2, Oriental Yeast Co., LTD., Japan) was given every day. Water; drinking water.
3. Health check was performed by stuffs every day before and after pacemaker implantation in both operated group and non-sham operated group. If necessary, animal doctors saw dogs and treated them.

In 6 adult beagle dogs (10–13 kg), heart failure was induced by continuous application of rapid right ventricular pacing at 250 bpm using an externally programmable miniature pacemaker (Medtronic Inc., Minneapolis, MN or Taisho Biomed Instruments Co., Ltd) for 28 days, as described previously [6, 24, 25]. Dogs were deeply anaesthetized with an isoflurane and intravenous injection of sodium pentobarbital (50mg/kg) so that a pacemaker lead could be inserted into the right ventricle apex via left jugular vein under fluoroscopy and connected to a pacemaker implanted subcutaneously in the neck. Six non-sham operated dogs were used as controls. Before sacrificing non-sham operated controls and 4weeks-pacing dogs, we measured heart rate, blood pressure, and indices of cardiac function by echocardiography in order to confirm that 4-weeks pacing induced heart failure (HF) under conscious condition. At the end of the study, dogs were euthanized with an isoflurane and intravenous injection of sodium pentobarbital and ventilated mechanically, followed by rapid removal of heart as previous described [6, 24, 25]. Hearts were rapidly excised via thoracotomy. These procedures were performed at an animal operation room of Science Research Center at Yamaguchi University. This study conforms to the Guide for the Care and Use of Laboratory Animals published by the US National Institutes of Health (NIH Publication No. 85-23, revised 1996). All animal protocols were approved by the Yamaguchi University School of Medicine Animal Experiment Committee (institutional permission # 23–027).

### Isolation of cardiomyocytes

Cardiomyocytes were isolated from the LV free wall of the beagles with a little modification as described previously [6, 24, 25]. Briefly, a wedge of the LV free wall perfused by a diagonal branch of left anterior descending coronary artery was resected from the whole heart and quickly perfused with perfusion buffer without collagenase (95%O_2_/5%CO_2_ -bubbled Minimal Essential Medium (Sigma) supplemented with 50 μM Ca^2+^, 0.5 mg/mL and 0.02 mg/mL protease type XIV). Then, antegrade perfusion from the coronary artery branch was performed for 1 hour with perfusion buffer with collagenase (95%O2/5%CO2 -bubbled Minimal Essential Medium (Sigma) supplemented with 50 μM Ca^2+^, 0.5 mg/mL collagenase B, 0.5 mg/mL, collagenase D and 0.02 mg/mL protease type XIV). The temperature of the perfusion buffer kept 37°C. Finally, the perfused LV was minced with scissors and rod-shaped adult canine cardiomyocytes were prepared. The Ca^2+^ concentration in the incubation medium was gradually increased to a final concentration of 1 mM (50μM, 125 μM, 300 μM, and 1 mM). The isolated cardiomyocytes were transferred to laminin-coated glass culture dishes and incubated for 12 h at 37°C in a 95% O_2_/5% CO_2_ atmosphere. It took 6 hours to finish the isolation of cardiomyocytes since the measurement of cardiac function and LV geometry by echocardiography. Measurement of cell shortening and Ca^2+^ transient, Ca^2+^ spark assay were started after 12 hour-incubation (overnight), and all measurements were finished within 8 hours.

### Measurement of cell shortening and Ca^2+^ transients

Cardiomyocyte cell shortening (CS) and intracellular Ca^2+^ transients (CaT) were measured using Fura-2 AM as described previously [6, 24, 25]. Briefly, cells were stimulated electrically by a field stimulator (IonOptix, MA) at a frequency of 0.5 Hz. CaT and CS amplitudes reached the steady state within 30 sec after start of pacing stimulation. Therefore, we recorded CaT and CS from 30 sec to 40 sec after start of pacing at the rate of 0.5 Hz. We defined the values of CaT peak and CS peak, which were calculated from averaging 10 consecutive steady CaT waveforms and 10 CS waveforms by using IonOptix analysis software, as the peak CaT and the peak CS of each cardiomyocyte. Ca^2+^-induced fluorescence at 505 nm was measured by excitation at 340 and 380 nm using a dual-excitation spectrofluorometer. The intracellular calcium concentration was calculated as the ratio of the fluorescence emission intensities at these 2 excitation wavelengths [6, 24, 25].

To determine the dose-dependent effect of landiolol on CS in isolated normal and failing cardiomyocytes, we measured CS with various doses of landiolol (from 0 nM to 1000 nM).

### Analysis of Ca^2+^ sparks with laser scanning confocal microscopy

Ca^2+^ sparks were measured as previously described [6, 24, 25, 26], using a laser scanning confocal microscope (LSM-510; Carl Zeiss) equipped with an argon ion laser and coupled to an inverted microscope (Axiovert 100, Carl Zeiss) with a Zeiss 40× oil-immersion Plan-Neofluor objective (1.3 numerical aperture; excitation at 488 nm; emission > 505 nm). Cardiomyocytes were loaded with 20 μM Fluo-4 AM (Molecular Probes) for 30 min at room temperature in the dark. Then, these cardiomyocytes were washed. Within 30 sec after start of pacing, CaT and CS amplitudes reached the steady state. Therefore, Ca^2+^ sparks were recorded from 30 sec to 40 sec after start of pacing at the rate of 0.5 Hz. Thus, Ca^2+^ spark frequency for each image (also for each group) was measured in the same scanning window to exclude the possibility that different Ca^2+^ spark frequency caused by different laser scanning time. Each cardiomyocyte was scanned repeatedly at 325.7 Hz along a line parallel to the longitudinal axis of the cell to avoid nuclei. The data were analyzed with SparkMaster, an automated analysis program that allows rapid and reliable Ca^2+^ spark analysis in confocal line-scan images, as described previously [6, 24, 25, 26].

### Measurement of intra-sarcoplasmic reticulum Ca^2+^ concentration in cardiomyocytes

A caffeine-induced Ca^2+^ transient was measured by first applying a stimulation train at 0.5 Hz for 60 sec and then rapidly switching the superfusion solution to a solution containing 20 mM caffeine for 5–6 s, as previously described [6, 24, 25, 26].

### Measurement of landiolol antioxidative effect on intact cardiomyocyte

In canine cardiomyocytes, a fluorescent probe, 2,7-dichlorofluorescin diacetate (DCFH-DA, Molecular Probes), was used to assess intracellular reactive oxygen species (ROS) formation, as described previously [27, 28]. Fluorescence images (excitation at 490 nm, emission at 530 nm) were acquired with a microscope (LSM 510, Carl Zeiss, Oberkochen, Germany).

### Immunoblot analysis

We performed immunoblot analyses using specific antibodies against ryanodine receptor 2 (RyR2; Sigma), Ser2808-phosphorylated RyR2 (P-Ser2808-RyR2; Badrilla), phospholamban (PLB; Upstate Biotech), Ser16-phosphorylated PLB (P-Ser16-PLB; Upstate Biotech), and Thr17-phosphorylated PLB (P-Thr17-PLB; Badrilla) as previously described [26, 29].

### Statistical analysis

The chi-squared test was used to compare prevalence or frequencies. The significance of differences between 2 groups was determined by post-hoc tests with Least Significant Difference algorithms following repeated-measures analysis of variance to evaluate the dose-dependence of landiolol on cell shortening in isolated cardiomyocytes. Comparisons across milrinone(+/-), landiolol(+/-), and heart failure(+/-) were independently verified with multivariate analysis of variance in experimental studies. Kruskal Wallis ANOVA was used to evaluate the antioxidative effect of landiolol on intact cardiomyocytes. All analyses were performed with SPSS 18.0 software (SPSS Inc., Chicago, Illinois). *P* values less than 0.05 were considered statistically significant.

## Results

### The comparison of hemodynamics in normal and heart failure model

After 4 weeks-rapid pacing, decreased left ventricular ejection fraction (LVEF), dilated left ventricular end-diastolic dimension (LVDD) and dilated left ventricular end-systolic dimension (LVDS) were confirmed in HF group as compared with non-sham operated controls (Table 1). There was no difference in heart rate (HR) and blood pressure between HF group and controls. These data were compatible with the hemodynamic data which were previously reported [5, 6, 24, 25, 27, 28, 30].

**Table 1:** Hemodynamic Data.

	**HR, bpm**	**SBP, mmHg**	**DBP, mmHg**	**LVDD, mm**	**LVDS, mm**	**LVFS, %**
Control (n = 6)	114 ± 26	135 ± 6	78 ± 7	31.2 ± 1.3	19.8 ± 1.5	36.3 ± 4.2
HF (n = 6)	118 ± 11	126 ± 12	68 ± 24	39.2 ± 1.7*	34.5 ± 1.9*	11.9 ± 3.8*

Control, non-sham operated control; HF, pacing-induced heart failure group; HR, heart rate; SBP, systolic blood pressure; DBP, diastolic blood pressure; LVDD, left ventricular end-diastolic diameter; LVDS, left ventricular end-systolic diameter; LVFS, left ventricular fractional shortening. Each datum point represents the mean ± SD. The number of experiments is shown in the parentheses. Unpaired T-test was employed to determine the statistical significance of the data (p value).

*p<0.05 vs Control.

### Effects of landiolol or milrinone on Ca^2+^ handling and cell function in isolated canine cardiomyocytes

**Figure 2:**
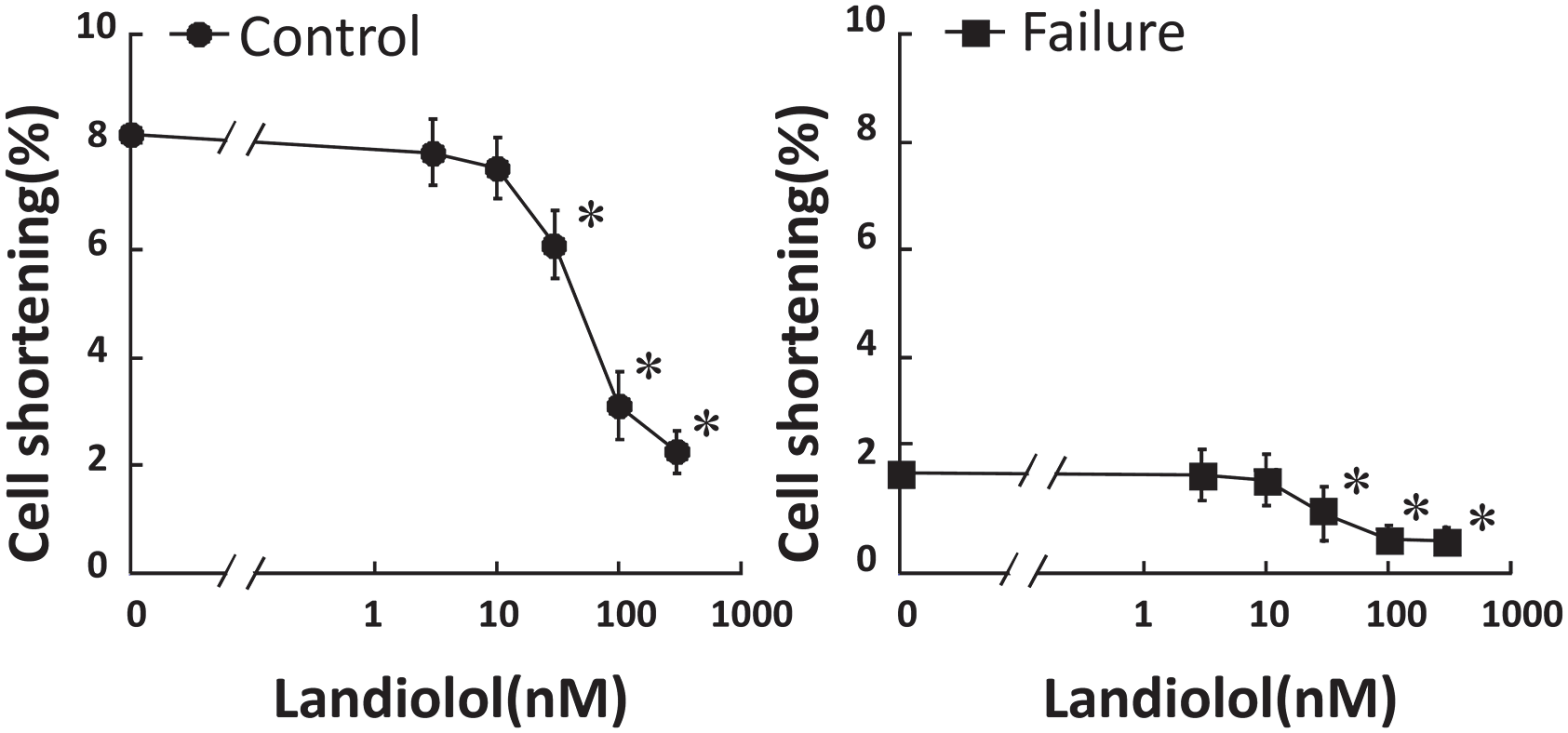
Dose-dependent inhibition of cell shortening by landiolol in normal and failing cardiomyocytes. Each group contained 20–30 cells. * *P*<0.05 vs. baseline.

**Figure 3:**
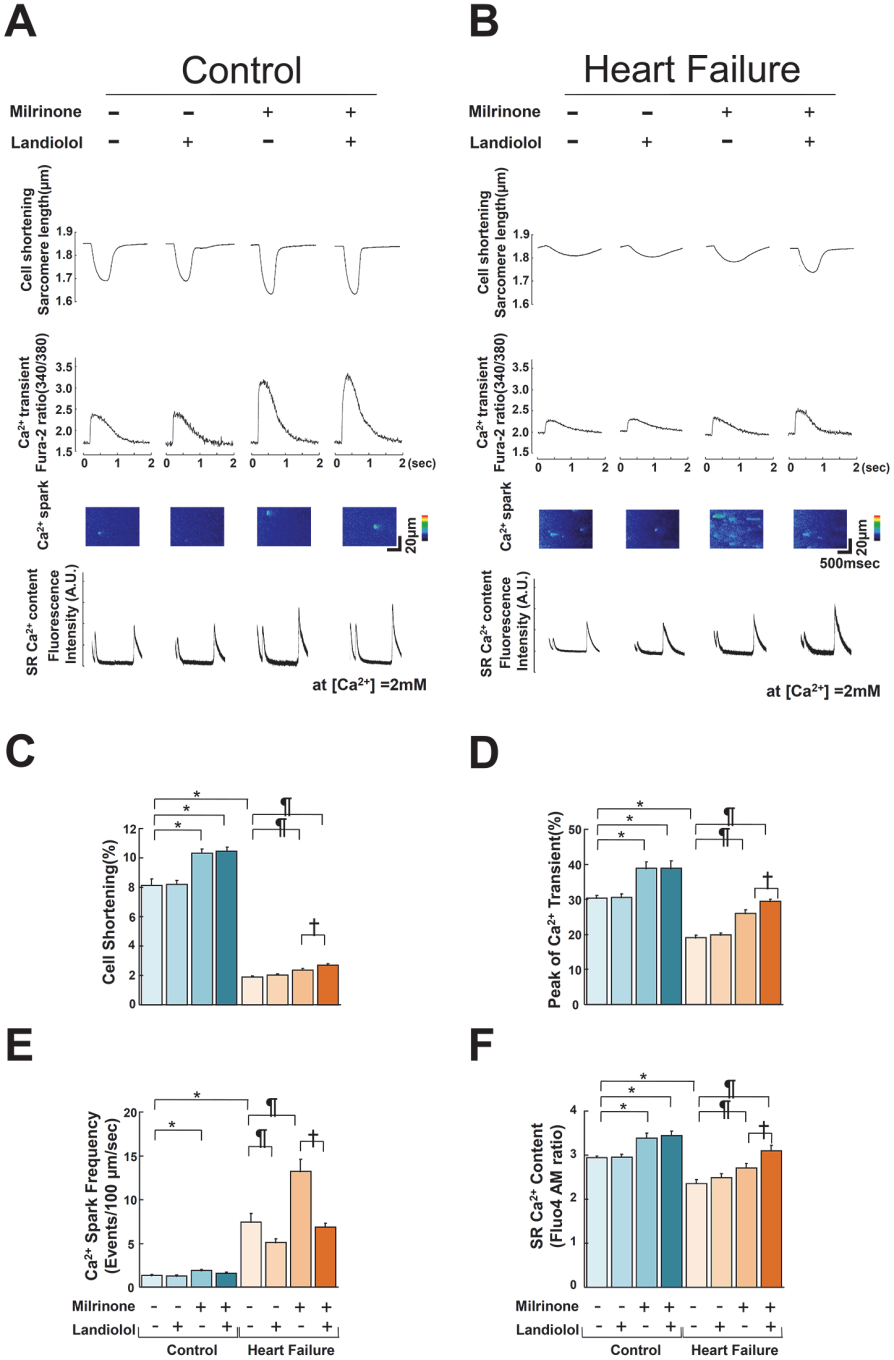
Effect of milrinone or landiolol on cell shortening, Ca^2+^ transient, Ca^2+^ spark, and sarcoplasmic reticulum Ca^2+^ concentration in normal and failing cardiomyocytes. A, B. Representative data for cell shortening, Ca^2+^ transient, diastolic Ca^2+^ spark, and SR Ca^2+^ content in control and failing cardiomyocytes. -, no treatment; +, 10 μM milrinone or 10 nM landiolol. C, D, E, F. A bar graph representation of the data in Fig. 3A, B. The bars indicate the mean (SE). Each group included 20–30 cells. At least 4 cells were evaluated for each preparation.* *P*<0.05 vs. control (baseline), ^¶^ *P*<0.05 vs. failure (baseline), ^†^ *P*<0.05 vs. failure (monotreatment with milrinone).

**Figure 4:**
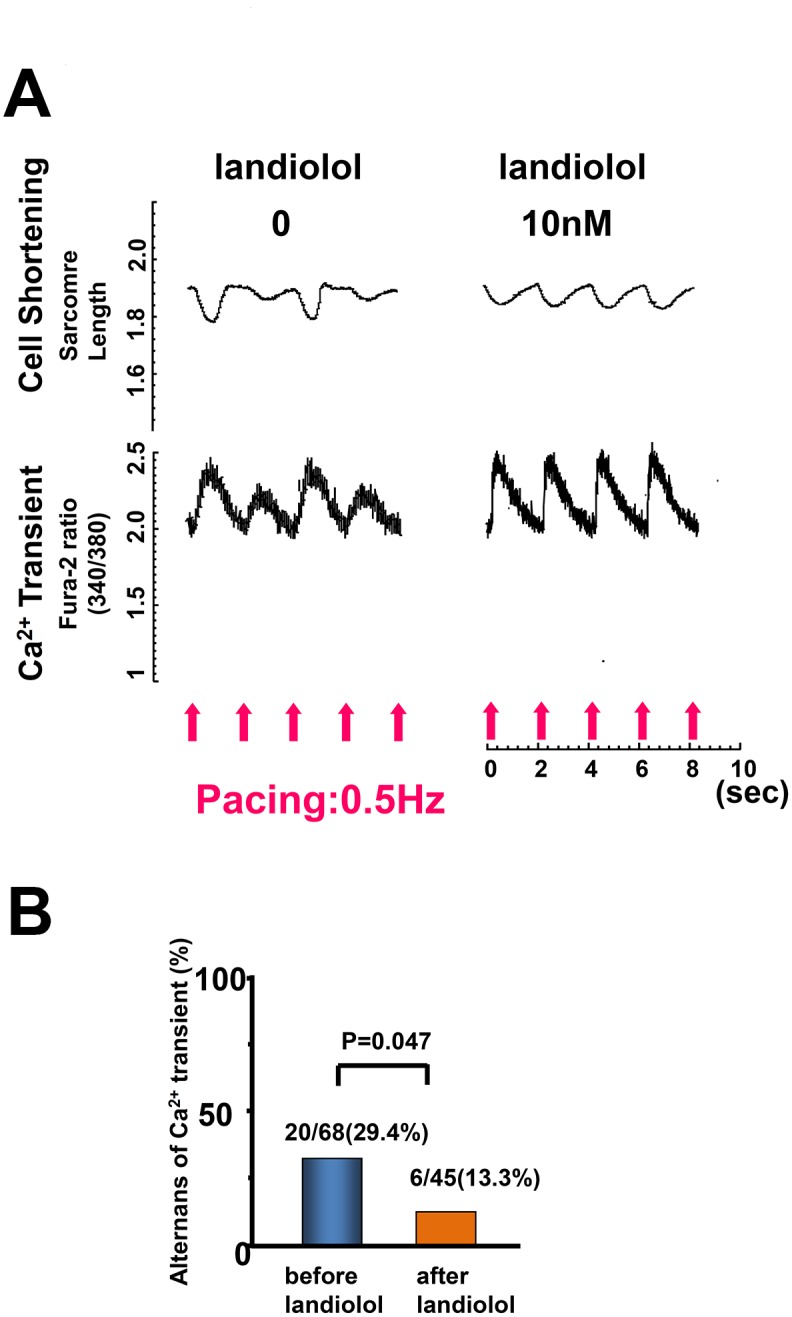
Alternans of cell shortening and Ca^2+^ transient in failing cardiomyocytes and its recovery by low-dose landiolol. A. Representative data. B. A bar graph representation of the data in Fig. 4A.

As shown in Fig. 2, the addition of less than 10 nM landiolol did not have any appreciable effect on CS in both normal and failing cardiomyocytes; however, more than 30 nM landiolol significantly inhibited CS. On the basis of these results, we defined 10 nM landiolol as the “low dose”. We also used 10 μM milrinone (maximum effect dose) for Ca^2+^ handling experiments, as described previously [31, 32]. In failing cardiomyocytes, the frequency of Ca^2+^ sparks (CaSF) increased significantly, and both peak CaT and CS decreased markedly compared with normal cardiomyocytes (Fig. 3A, B). The addition of 10 μM milrinone to failing cardiomyocytes significantly increased peak CaT, peak CS, CaSF, and {Ca^2+^}_SR_. Interestingly, the co-addition of landiolol and milrinone to failing cardiomyocytes largely decreased the milrinone-enhanced CaSF, and in turn, significantly increased {Ca^2+^}_SR_, peak CaT and peak CS as compared with milrinone mono-treatment in failing cardiomyocytes. In addition, low-dose landiolol significantly inhibited the alternans of Ca^2+^ transient and CS under a fixed pacing rate (0.5 Hz) in failing cardiomyocytes (P = 0.047; Fig. 4A, B).

### Effect of low-dose landiolol on the phosphorylation of cardiac ryanodine receptor 2 and phospholamban

**Figure 5:**
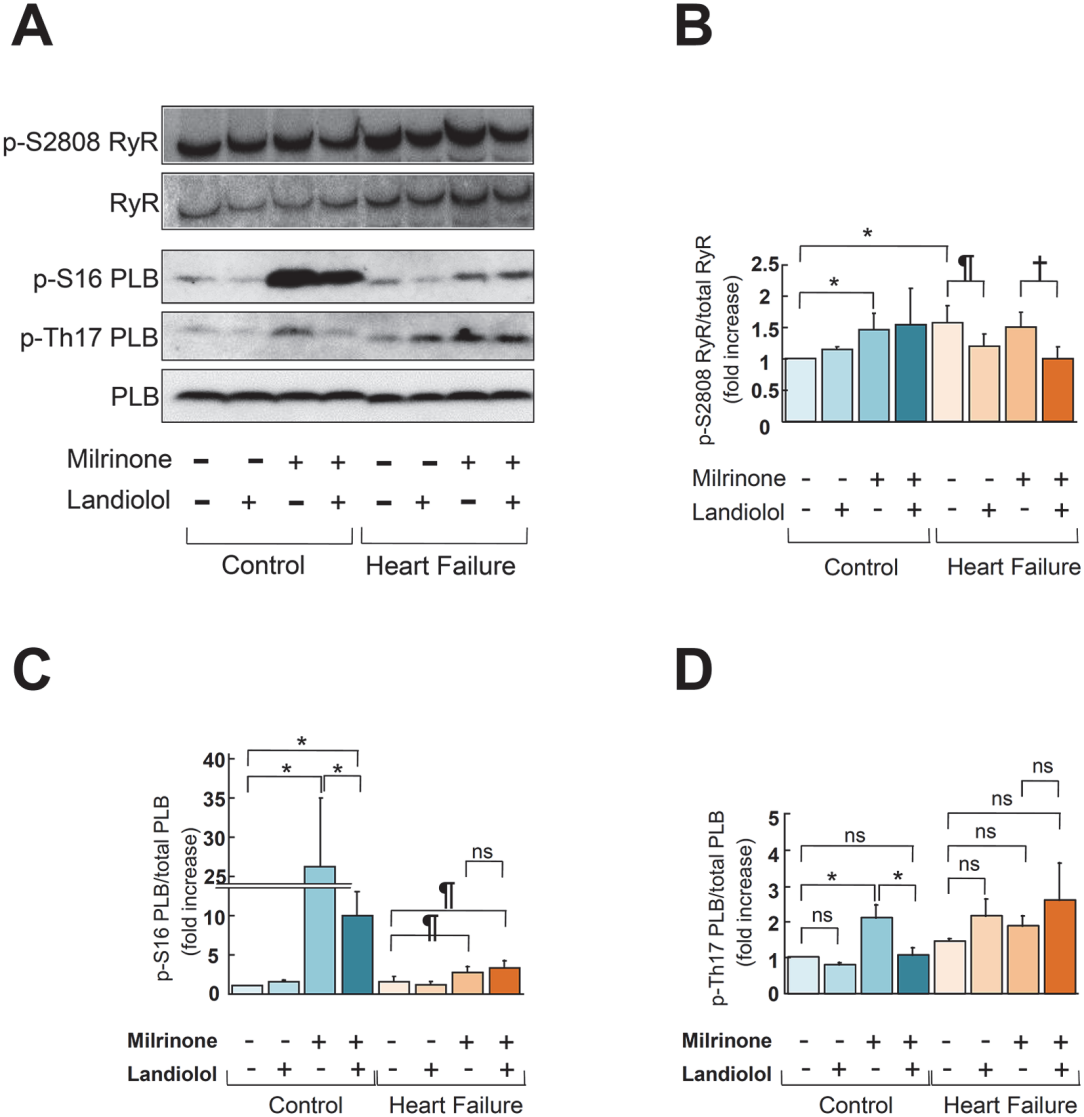
Immunoblots of phosphorylated RyR (Ser2808), total RyR2, phosphorylated PLB (Ser16, Thr17), and total PLB in normal and failing cardiomyocytes. A. Representative data. B, C, D. The corresponding bar graphs, with bars indicating the mean (SE). The results of the quantitative analysis are expressed relative to the control (baseline) value, which was designated as 1 (n = 6 in each group). * *P*<0.05 vs. control (baseline), ^¶^ *P*<0.05 vs. failure (baseline), ^†^ *P*<0.05 vs. failure (monotherapy with milrinone).

In normal cardiomyocytes, milrinone (10 μM) slightly increased the phosphorylation levels of RyR2, Ser2808, and PLB Thr17 and markedly increased that of PLB Ser16 (Fig. 5A, B, C, D). The addition of low-dose landiolol to milrinone suppressed PLB phosphorylation without any appreciable effect on RyR2 phosphorylation (Fig. 5A, B, C, D).

In failing cardiomyocytes, the baseline RyR2 phosphorylation level was abnormally elevated, as described previously [5, 33, 34]. Milrinone (10 μM) had no additional effect on the hyperphosphorylation of RyR2 Ser2808 but significantly increased the phosphorylation of PLB Ser16 and Thr17 (Ser16 > Thr17). Low-dose landiolol suppressed RyR2 hyperphosphorylation but had no effect on PLB phosphorylation in the presence or absence of milrinone (Fig. 5A, B, C, D).

### Measurement of landiolol antioxidative effect on intact cardiomyocytes

**Figure 6:**
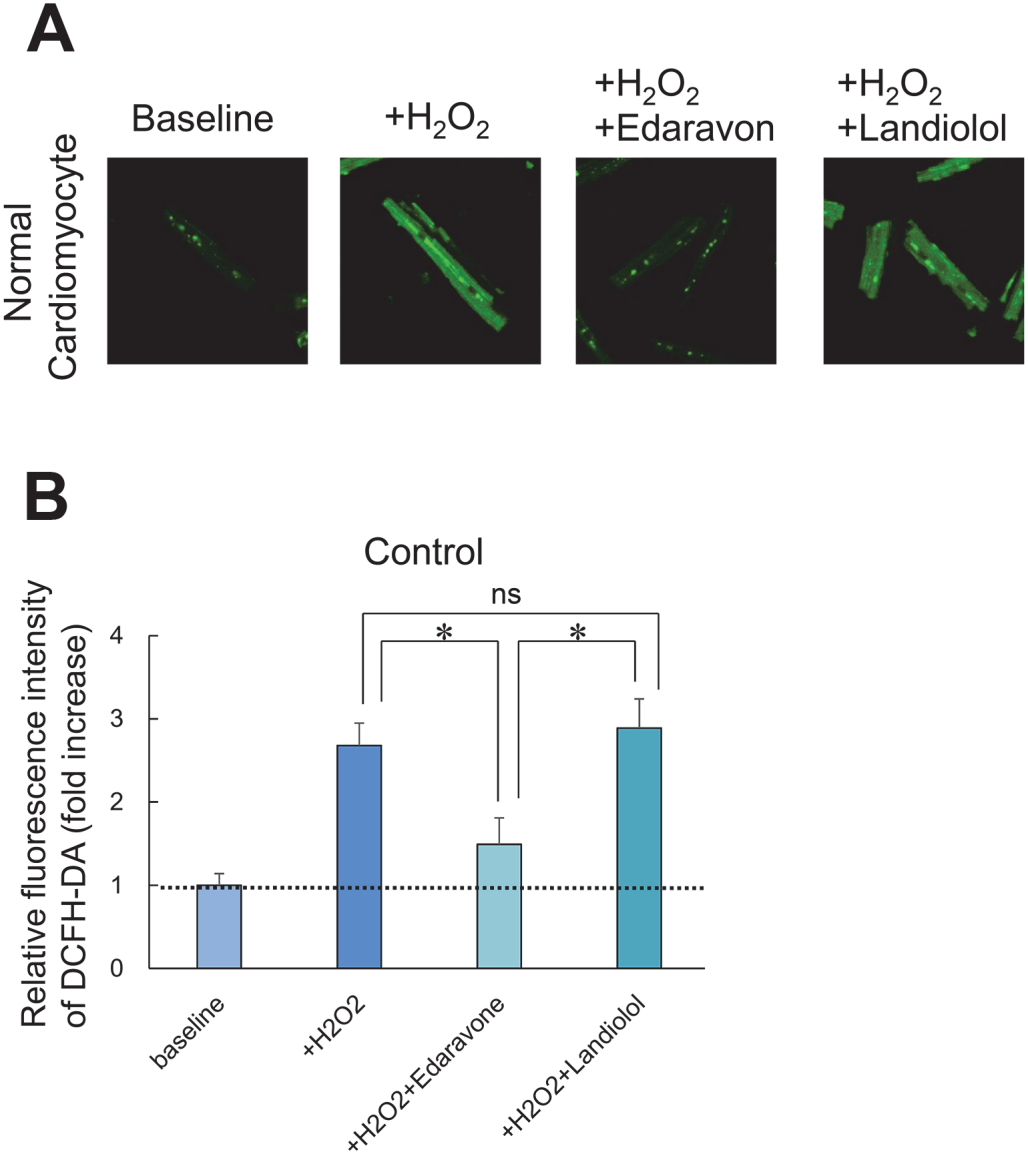
Antioxidative effect of landiolol on intact cardiomyocytes. Representative data. In normal cardiomyocytes, fluorescence intensity of DCFH-DA was significantly increased after addition of 100μmol/L H_2_O_2_ and restored to a normal level in the presence of 100μmol/L edaravone, while it remained increased in the presence of 10 nmol/L landiolol.

Fig. 6 shows fluorescence images after application of a fluorescent probe of intracellular ROS, DCFH-DA (1 μmol/L), to normal cardiomyocytes. In normal cardiomyocytes, fluorescence intensity was markedly increased after addition of 100 μM H_2_O_2_, whereas it was restored to normal levels in the presence of 100 μM edaravone, which is a radical scavenger. By contrast, fluorescence intensity was not altered in the presence of 10 nmol/L landiolol. (Fig. 6A, B).

## Discussion

The most important new aspects of the present study are the findings that 1) landiolol, a pure β1-blocker, inhibited Ca^2+^ leakage from failing RyR2 even at a low dose that did not suppress cardiomyocyte function; 2) milrinone monotherapy enhanced Ca^2+^ leakage from failing RyR2, while adding low-dose β1-blocker to milrinone suppressed this milrinone-induced Ca^2+^ leakage, leading to greater improvement in cardiomyocyte function; and 3) low-dose landiolol prevented mechanical alternans in failing myocardiocytes. This report is the first to demonstrate that a low-dose pure β1-blocker in combination with milrinone can acutely benefit abnormal intracellular Ca^2+^ handling. Our results (Fig. 3A–F) suggest the following mechanism: milrinone alone slightly elevates {Ca^2+^}_SR_ and peak CaT by a net effect of enhanced Ca^2+^ uptake through PLB phosphorylation and Ca^2+^ leakage through hyperphosphorylated RyR2. The addition of low-dose landiolol to milrinone suppresses RyR2 hyperphosphorylation and therefore stops Ca^2+^ leakage, which in turn further increases {Ca^2+^}_SR_ and peak CaT, leading to markedly improved cell function (Fig. 3A–F).

We previously reported the first observation that pulsus alternans, a well-known sign of severe heart failure, was completely eliminated by addition of low-dose landiolol in 10 patients with severe ADHF [15]. The mechanism of this effect remains unclear. Pulsus alternans is more likely to occur at higher heart rates [35], and the heart rate reduction achieved by a low-dose β1-blocker may be involved in eliminating it. However, several studies have shown that pulsus alternans arises from abnormal intracellular calcium cycling involving SR [22, 23]. Therefore, we hypothesized that low-dose β1-blocker also corrects abnormal intracellular Ca^2+^ handling during heart failure. To test this hypothesis, we examined the effect of low-dose landiolol on Ca^2+^ release through RyR2 and CS by electrically pacing isolated cardiomyocytes. Alternans of Ca^2+^ transient and cell shortening appeared in 30% of intact failing cardiomyocytes, and not at all in intact normal cardiomyocytes. Addition of low-dose landiolol significantly diminished the alternans of Ca^2+^ transient and CS (Fig. 4A, B). These findings strongly imply that this β1-blocker improved aberrant intracellular Ca^2+^ handling irrespective of heart rate.

One of the major regulators of cardiac contractility is 3′-5′-cyclic adenosine monophosphate (cAMP)-dependent protein kinase A (PKA) phosphorylation via β-adrenergic stimulation [2, 5, 33, 34]. However, in chronic heart failure, intracellular Ca^2+^ overload and Ca^2+^ depletion in SR are due not only to Ca^2+^ leakage from failing RyR2 but also to decreased Ca^2+^ uptake, which is caused by down-regulation of sarcoma/endoplasmic reticulum Ca^2+^-ATPase and decreased PLB phosphorylation [2, 5, 33, 34]. A low-dose β1-blocker that induced dephosphorylation of both RyR2 and PLB would worsen cardiomyocyte function, not, as we observed, improve it. To determine the molecular mechanism of the observed effects, we examined the effect of milrinone (10 μM) or low-dose landiolol (10 nM) on RyR2 and PLB phosphorylation in normal and failing cardiomyocytes. Our results suggest that a low-dose β1-selective blocker inhibits Ca^2+^ leakage through RyR2 by selectively suppressing RyR2 phosphorylation during heart failure (Fig. 5A, B). Therefore, combination therapy with milrinone and low-dose landiolol might be a superior therapeutic strategy for ADHF because it improves cardiomyocyte function and prevents lethal arrhythmia resulting from intracellular Ca^2+^ overload.

In heart failure, the difference in phosphorylation level between RyR2 and PLB might arise from the compartmentation of the PKA signaling cascade [36–40]. Indeed, our results showed that milrinone promoted PLB Ser16 and Thr17 (but not RyR2 Ser2808) phosphorylation in failing cardiomyocytes, while low-dose landiolol inhibited RyR2 Ser2808 hyperphosphorylation (but not milrinone-induced PLB Ser16 and Thr17 phosphorylation). Taken together, these findings indicate that inhibition of aberrant Ca^2+^leakage through failing RyR2, which was enhanced by milrinone, with a low-dose β1-blocker might improve cardiac function and suppress arrhythmogenesis [1, 2, 15]

Tachycardia itself complicated acute heart failure-induced intracellular Ca^2+^ overload and enhanced myocardial oxidative stress [41]. Therefore, slowing HR with a β1-blocker is considered cardioprotective. In the present study, however, the cardioprotective effect occurred through inverse agonism of the β1-blocker independent of HR, as all functional experiments were performed at steady rate of 0.5 Hz pacing and in the absence of catecholamine. Based on the present results, milrinone-induced lethal arrhythmia appears to be associated with enhanced diastolic Ca^2+^ leakage from SR. Therefore, low-dose landiolol in combination with milrinone may be a novel strategy to prevent lethal arrhythmia in patients with acute heart failure.

Another important mechanism of abnormal diastolic Ca^2+^ release through RyR2 is the oxidation of RyR2 due to ROS [27, 28]. In the present study, however, landiolol had no appreciable antioxidant effect on cardiomyocytes in the presence of 100 μmol/L H_2_O_2_ (Fig. 6A, B). Therefore, the antioxidant effect of landiolol does not appear to contribute to suppressing diastolic Ca^2+^ leakage from SR.

While β1 adrenergic receptor (β1AR) blocker plays a role through its blocking β1AR, the model used in the present study is the cultured cells where there is no any catecholamine in the medium. How does the β1AR play the role in regulation of intracellular Ca^2+^ homeostasis? In the present study, it was suggested that the inverse agonism of landiolol via β1AR, but not its competitive inhibition with catecholamines, contributed to the mechanism by which landiolol inhibited diastolic Ca^2+^ leakage from RyR2 by the selective inhibition of phosphorylation of RyR2 in failing cardiomyocytes. It was reported that β blockers such as nebivolol, bisoprolol, metoprorol, carvediolol, and bucindolol had inverse agonism effect in human ventricular or atrial myocardium [42].

Are the phenomena which landiolol induced, landiolol-specific? Other β blockers might have similar effects to greater or lesser degree. The reasons are as follows; 1) β blockers such as nebivolol, bisoprolol, metoprorol, carvediolol, and bucindolol have inverse agonism effect [42], 2) β blockers such as propranolol and carvedilol suppress Ca^2+^ leak from SR in failing cardiomyocytes [27, 33].

**Figure 7:**
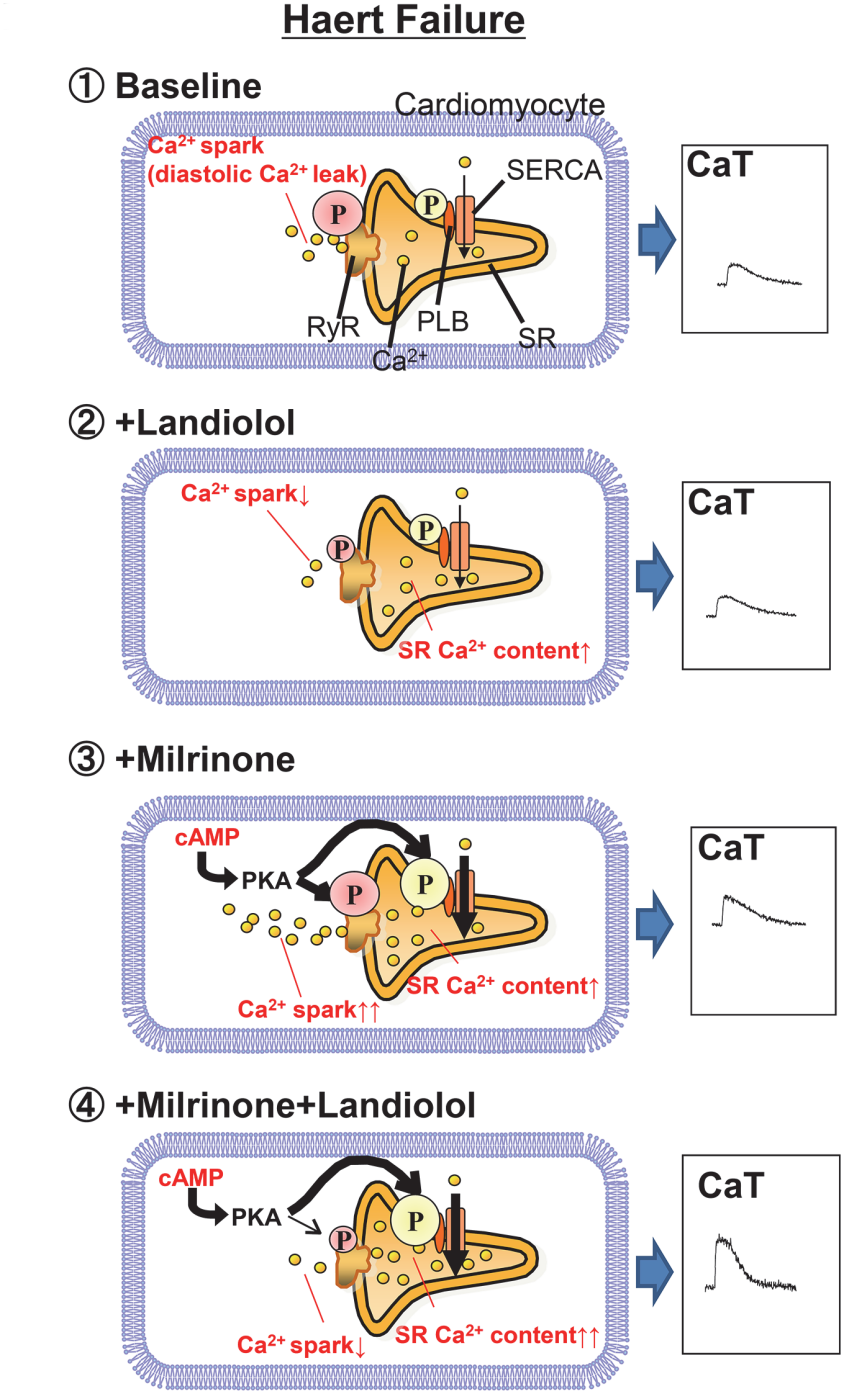
Proposed mechanism of inhibition of milrinone-induced Ca^2+^ sparks (Ca^2+^ leakage) from the sarcoplasmic reticulum.

On the basis of our results, we propose the following model for the molecular basis of low-dose β-blocker treatment of ADHF (Fig. 7). First, in the baseline condition, enhanced phosphorylation of RyR2 Ser2808 induces Ca^2+^ leakage from SR, which causes intracellular Ca^2+^ overload and decreases {Ca^2+^}_SR_. Second, a low-dose β1-blocker selectively suppresses RyR2 Ser2808 hyperphosphorylation to inhibit Ca^2+^ leakage from SR but leave Ca^2+^ uptake through the sarco/endoplasmic reticulum Ca^2+^-ATPase unchanged. Third, monotherapy with milrinone selectively increases phosphorylation of PLB Ser16 and Thr17, but not to the extent of RyR2 Ser2808. Additionally, Ca^2+^ leakage from SR increases proportionally to increasing Ca^2+^ uptake. Eventually, the peak Ca^2+^ transient is slightly elevated. Fourth, combination therapy with milrinone and a low-dose β-blocker increases phosphorylation of PLB Ser16 and Thr17 and suppresses that of RyR2 Ser2808. These drugs also increase Ca^2+^ uptake and decrease Ca^2+^ leakage, which increases {Ca^2+^}_SR_ and the peak Ca^2+^ transient.

### Limitations

Inhibition of milrinone-induced diastolic Ca^2+^ leakage from the failing SR has been suggested to arise in part from selective inhibition of phosphorylated RyR2 (Ser 2808), the target amino acid of cAMP-dependent PKA. In the present study, however, we did not directly examine the effect of low-dose landiolol on phosphorylation of RyR2 (Thr 2814), the target amino acid of Ca^2+^/calmodulin-dependent protein kinase II (CaMK II). Recently, several reports indicated that CaMK II, rather than PKA, plays a critical role in diastolic Ca^2+^ leak through RyR2 [43, 44]. Therefore, the mechanism by which low-dose landiolol suppressed milrinone-induced diastolic Ca^2+^ leak may also involve inhibition of RyR2 (Thr 2814) phosphorylation.

The phosphorylation level for PLB-Ser16 (PKA phosphorylated site) is much larger than PLB-Thr17 (CaMKII phosphorylated site) after addition of milrinone, which may suggest that milrinone affects Ca^2+^ handling through PKA phosphorylated site. Xiao B et al. reported that RyR2-Ser2030 site was the major phosphorylation site in RyR2 responding to PKA activation upon β adrenergic stimulation in normal and failing rat hearts [45]. In the present study, however, we did not investigate the effect of milrinone and/or landiolol on the phosphorylation level of RyR2-Ser2030 in dog cardiomyocytes. Therefore, the mechanism by which low-dose landiolol suppressed Ca^2+^ leakage through RyR2 may be due to the inhibition of phosphorylation of RyR2-Ser2030 as well as the inhibition of phosphorylation of RyR2-Ser2808. Further research is needed to clarify these possibilities.

## Conclusions

In failing cardiomyocytes, the addition of a low-dose β1-blocker to milrinone improved intracellular Ca^2+^ handling and significantly restored mechanical alternation by inhibiting diastolic Ca^2+^ leakage from SR. Thus, the molecular mechanism by which a low-dose β1-blocker can suppress milrinone-induced Ca^2+^ leakage from SR is very important for the treatment of ADHF.

## Supporting Information

S1 ARRIVE Checklist Supporting information is available in the ARRIVE checklist. (DOC)

## References

[1] Dobrev D, Wehrens XH. (2014) Role of RyR2 phosphorylation in heart failure and arrhythmias: controversies around ryanodine receptor phosphorylation in cardiac disease. Circ Res 114:1311–1319. 10.1161/CIRCRESAHA.114.300568 24723656 PMC4008932

[2] Marks AR. (2013) Calciumcycling proteins and heart failure: mechanisms and therapeutics. J Clin Invest 123:46–52. 10.1172/JCI62834 23281409 PMC3533269

[3] McCauley MD, Wehrens XH. (2011) Targeting ryanodine receptors for anti-arrhythmic therapy. Acta Pharmacol Sin 32:749–757. 10.1038/aps.2011.44 21642946 PMC4009959

[4] Yano M, Ikeda Y, Matsuzaki M. (2005) Altered intracellular Ca2+ handling in heart failure. J Clin Invest 115:556–564. 10.1172/JCI24159 15765137 PMC1052007

[5] Yano M, Kobayashi S, Kohno M, Doi M, Tokuhisa T, et al (2003) FKBP12.6-mediated stabilization of calcium-release channel (ryanodine receptor) as a novel therapeutic strategy against heart failure. Circulation 107:477–484. 10.1161/01.CIR.0000044917.74408.BE 12551874

[6] Kobayashi S, Yano M, Suetomi T, Ono M, Tateishi H, et al (2009) Dantrolene, a therapeutic agent for malignant hyperthermia, markedly improves the function of failing cardiomyocytes by stabilizing inter-domain interactions within the ryanodine receptor. J Am Coll Cardiol 53:1993–2005. 10.1016/j.jacc.2009.01.065 19460614 PMC2764410

[7] Majure DT, Teerlink JR. (2011) Update on the management of acute decompensated heart failure. Curr Treat Options Cardiovasc Med 13:570–585. 10.1007/s11936-011-0149-2 21976129

[8] Thomas SS, Nohria A. (2012) Hemodynamic classifications of acute heart failure and their clinical application: – an update –. Circ J 76:278–286. 10.1253/circj.CJ-11-1441 22240601

[9] Lowes BD, Tsvetkova T, Eichhorn EJ, Gilbert EM, Bristow MR. (2001) Milrinone versus dobutamine in heart failure subjects treated chronically with carvedilol. Int J Cardiol 81:141–149. 10.1016/S0167-5273(01)00520-4 11744130

[10] Mann DL, Kent RL, Parsons B, Cooper G 4th. (1992) Adrenergic effects on the biology of the adult mammalian cardiocyte. Circulation 85:790–804. 10.1161/01.CIR.85.2.790 1370925

[11] Maytin M, Colucci WS. (2005) Cardioprotection: a new paradigm in the management of acute heart failure syndromes. Am J Cardiol 96:26G–31G. 10.1016/j.amjcard.2005.07.018 16181820

[12] Yoshida Y, Hongo T, Sakamoto A, Ogawa R. (2005) Successful management of tachycardia atrial fibrillation in a septic patient with landiolol. Anesth Analog 100:294–295. 10.1213/01.ANE.0000140814.28118.6F 15616100

[13] Yoshida Y, Terajima K, Sato C. (2008) Clinical role and efficacy of landiolol in the intensive care unit. J Anesth 22:64–69. 10.1007/s00540-007-0573-3 18306018

[14] Ikeshita K, Nishikawa K, Toriyama S, Yamashita T, Tani Y, et al (2008) Landiolol has a less potent negative inotropic effect than esmolol in isolated rabbit hearts. J Anesth 22:361–366. 10.1007/s00540-008-0640-4 19011773

[15] Kobayashi S, Susa T, Tanaka T, Murakami W, Fukuta S, et al (2012) A low-dose β blocker in combination with milrinone safely improves cardiac function and eliminates pulsus alternans in patients with acute decompensated heart failure. Circ J 76:1646–1653. 10.1253/circj.CJ-12-0033 22481100

[16] Inoue H, Arata H, Okumura K, Kamakura S, Kumagai K, et al. Guideline for pharmacotheraphy of atrial fibrillation (JCS2013). http://www.j-circ.or.jp/guideline/pdf/JCS2013_inoue_h.pdf (in Japanese).

[17] Nagai R, Kinugawa K, Inoue H, Atarashi H, Seino Y, et al; J-Land Investigators (2013) Urgent management of rapid heart rate in patients with atrial fibrillation/flutter and left ventricular dysfunction: comparison of the ultra-short-acting β1-selective blocker landiolol with digoxin (J-Land Study). Circ J 77:908–916. 10.1253/circj.CJ-12-1618 23502991

[18] Kobayashi S, Murakami W, Myoren T, Tateishi H, Okuda S, et al (2014) A low-dose β1 blocker effectively and safely slows heart rate in patients with acute decompensated heart failure and rapid atrial fibrillation. Cardiology 127:105–113. 10.1159/000355312 24296610

[19] Ryan JM, Schive JF, Hull HB, Oser BM. (1956) Experiences with pulsus alternans. Ventricular alternation and the stage of heart failure. Circulation 14:1099–1103. 10.1161/01.CIR.14.6.1099 13383806

[20] Mulieri LA, Hasenfuss G, Leavitt B, Allen PD, Alpert NR. (1992) Altered myocardial force-frequency relation in human heart failure. Circulation 85:1743–1750. 10.1161/01.CIR.85.5.1743 1572031

[21] Hasenfuss G, Reinecke H, Studer R, Pieske B, Meyer M, et al (1996) Calcium cycling proteins and force-frequency relationship in heart failure. Basic Res Cardiol 91 Suppl 2:17–22. 10.1007/BF00795357 8957539

[22] Kihara Y, Morgan JP. (1991) Abnormal Cai2+ handling is the primary cause of mechanical alternans: study in ferret ventricular muscles. Am J Physiol 261:H1746–1755. 1750531 10.1152/ajpheart.1991.261.6.H1746

[23] Györke S, Carnes C. (2008) Dysregulated sarcoplasmic reticulum calcium release: potential pharmacological target in cardiac disease. Pharmacol Ther 119:340–354. 10.1016/j.pharmthera.2008.06.002 18675300 PMC2798594

[24] Oda T, Yano M, Yamamoto T, Tokuhisa T, Okuda S, et al (2005) Defective regulation of interdomain interactions within the ryanodine receptor plays a key role in the pathogenesis of heart failure. Circulation 111:3400–3410. 10.1161/CIRCULATIONAHA.104.507921 15967847

[25] Yamamoto T, Yano M, Xu X, Uchinoumi H, Tateishi H, et al (2008) Identification of target domains of the cardiac ryanodine receptor to correct channel disorder in failing hearts. Circulation 117:762–772. 10.1161/CIRCULATIONAHA.107.718957 18227387

[26] Uchinoumi H, Yano M, Suetomi T, Ono M, Xu X, et al (2010) Catecholaminergic polymorphic ventricular tachycardia is caused by mutation-linked defective conformational regulation of the ryanodine receptor. Circ Res 106:1413–1424. 10.1161/CIRCRESAHA.109.209312 20224043 PMC2862146

[27] Mochizuki M, Yano M, Oda T, Tateishi H, Kobayashi S, et al (2007) Scavenging free radicals by low-dose carvedilol prevents redox-dependent Ca2+ leak via stabilization ofryanodinereceptor in heart failure.J Am Coll Cardiol 49:1722–1732. 10.1016/j.jacc.2007.01.064 17448375

[28] Yano M, Okuda S, Oda T, Tokuhisa T, Tateishi H, Mochizuki M, et al (2005) Correction of defective interdomain interaction within ryanodine receptor by antioxidant is a new therapeutic strategy against heart failure. Circulation 112:3633–43. 10.1161/CIRCULATIONAHA.105.555623 16330705

[29] Yamada M, Ikeda Y, Yano M, Yoshimura K, Nishino S, et al (2006) Inhibition of protein phosphatase 1 by inhibitor-2 gene delivery ameliorates heart failure progression in genetic cardiomyopathy. FASEB J 20:1197–1199. 10.1096/fj.05-5299fje 16627625

[30] Armstrong PW, Stopps TP, Ford SE, de Bold AJ. Rapid ventricular pacing in the dog: pathophysiologic studies of heart failure. (1986) Circulation 74:1075–1084. 10.1161/01.CIR.74.5.1075 2945673

[31] Endoh M, Yanagisawa T, Taira N, Blinks JR. (1986) Effect of new inotropic agents on cyclic nucleotide metabolism and calcium transients in canine ventricular muscle. Circulation 73: III-117–133. 2417745

[32] Gwathmey JG, Morgan JP. (1985) The effects of milrinone and piroximone on intracellular calcium handling in working myocardium from the ferret. Br J Pharmacol 85:97–108. 10.1111/j.1476-5381.1985.tb08835.x 2992656 PMC1916763

[33] Doi M, Yano M, Kobayashi S, Kohno M, Tokuhisa T, et al (2002) Propranolol prevents the development of heart failure by restoring FKBP12.6-mediated stabilization of ryanodine receptor. Circulation 105:1374–1379. 10.1161/hc1102.105270 11901051

[34] Marx SO, Reiken S, Hisamatsu Y, Jayaraman T, Burkhoff D, et al (2000) PKA phosphorylation dissociates FKBP12.6 from the calcium release channel (ryanodine receptor): defective regulation in failing hearts. Cell 101:365–376. 10.1016/S0092-8674(00)80847-8 10830164

[35] Hirashiki A, Izawa H, Somura F, Obata K, Kato T, et al (2006) Prognostic value of pacing-induced mechanical alternans in patients with mild-to-moderate idiopathic dilated cardiomyopathy in sinus rhythm. J Am Coll Cardiol 47:1382–1389. 10.1016/j.jacc.2005.10.069 16580526

[36] Fischmeister R, Castro LR, Abi-Gerges A, Rochais F, Jurevicius J, et al (2006) Compartmentation of cyclic nucleotide signaling in the heart: the role of cyclic nucleotide phosphodiesterases. Circ Res 99:816–828. 10.1161/01.RES.0000246118.98832.04 17038651

[37] Dodge-Kafka KL, Langeberg L, Scott JD. (2006) Compartmentation of cyclic nucleotide signaling in the heart: the role of A-kinase anchoring proteins. Circ Res 98: 993–1001. 10.1161/01.RES.0000218273.91741.30 16645149

[38] Zaccolo M, Movsesian MA. (2007) cAMP and cGMP signaling cross-talk: role of phosphodiesterases and implications for cardiac pathophysiology. Circ Res 100:1569–1578. 10.1161/CIRCRESAHA.106.144501 17556670

[39] Kapiloff MS. (2002) Contributions of protein kinase A anchoring proteins to compartmentation of cAMP signaling in the heart. Mol Pharmacol 62:193–199. 10.1124/mol.62.2.193 12130668

[40] Steinberg SF, Brunton LL. (2001) Compartmentation of G protein-coupled signaling pathways in cardiac myocytes. Annu Rev Pharmacol Toxicol 41:751–773. 10.1146/annurev.pharmtox.41.1.751 11264475

[41] Baartscheer A, Schumacher CA, Belterman CN, Coronel R, Fiolet JW. (2003) SR calcium handling and calcium after-transients in a rabbit model of heart failure. Cardiovasc Res 58:99–108. 10.1016/S0008-6363(02)00854-4 12667950

[42] Maack C, Tyroller S, Schnabel P, Cremers B, et al (2001) Characterization of beta(1)-selectivity, adrenoceptor-G(s)-protein interaction and inverse agonism of nebivolol in human myocardium. Br J Pharmacol 132:1817–26. 10.1038/sj.bjp.0703992 11309254 PMC1572729

[43] Zhang T, Maier LS, Dalton ND, Miyamoto S, Ross J Jr, et al (2003) The delta C isoform of CaMKII is activated in cardiac hypertrophy and induces dilated cardiomyopathy and heart failure. Circ Res 92:912–919. 10.1161/01.RES.0000069686.31472.C5 12676814

[44] Maier LS, Zhang T, Chen L, DeSantiago J, Brown JH, Bers DM. (2003) Transgenic CaMKII delta C overexpression uniquely alters cardiac myocyte Ca2+ handling: reduced SR Ca2+ load and activated SR Ca2+ release. Circ Res 92:904–911. 10.1161/01.RES.0000069685.20258.F1 12676813

[45] Xiao B, Zhong G, Obayashi M, Yang D et al Ser-2030, but not Ser-2808, is the major phosphorylation site in cardiac ryanodine receptors responding to protein kinase A activation upon beta-adrenergic stimulation in normal and failing hearts. (2006) Biochem J 396:7–16. 10.1042/BJ20060116 16483256 PMC1449991

