# Supplementary material for: A Low-Dose β1-Blocker in Combination with Milrinone Improves Intracellular Ca2+ Handling in Failing Cardiomyocytes by Inhibition of Milrinone-Induced Diastolic Ca2+ Leakage from the Sarcoplasmic Reticulum
Source: PLoS One. 2015 Jan 23;10(1):e0114314. doi: 10.1371/journal.pone.0114314 (PMC4304815; doi:10.1371/journal.pone.0114314)
Supplement: S1 ARRIVE Checklist — (DOC) [file pone.0114314.s001.doc]

**The ARRIVE checklist**

**TITLE**

**1 Provide as accurate and concise a description of the content of the article as possible.**

A low-dose β1-blocker in combination with milrinone improves intracellular Ca^2+^ handling in failing cardiomyocytes by inhibition of milrinone-induced diastolic Ca^2+^ leakage from the sarcoplasmic reticulum

**ABSTRACT**

**2 Provide an accurate summary of the background, research objectives (including details of the species or strain of animal used), key methods, principal findings, and conclusions of the study.**

**Background:** The molecular mechanism underlying how the combination of low-dose β1-blocker and milrinone affects intracellular Ca^2+^ handling in heart failure remains unclear. **Objectives:** The purpose of this study was to investigate whether adding a low-dose β1-blocker to milrinone improves cardiac function in failing cardiomyocytes and the underlying cardioprotective mechanism. **Methods:** We investigated the effect of milrinone plus landiolol on intracellular Ca^2+^ transient (CaT), cell shortening (CS), the frequency of diastolic Ca^2+^ sparks (CaSF), and sarcoplasmic reticulum Ca^2+^ concentration ([Ca^2+^]_SR_) in normal and failing canine cardiomyocytes and used immunoblotting to determine the phosphorylation level of ryanodine receptor (RyR2) and phospholamban (PLB). **Results:** In failing cardiomyocytes, CaSF significantly increased, and peak CaT and CS markedly decreased compared with normal myocytes. Administration of milrinone alone slightly increased peak CaT and CS, while CaSF greatly increased with a slight increase in [Ca^2+^]_SR_. Co-administration of β1-blocker landiolol to failing cardiomyocytes at a dose that does not inhibit cardiomyocyte function significantly decreased CaSF with a further increase in [Ca^2+^]_SR_, and peak CaT and CS improved compared with milrinone alone. Landiolol suppressed the hyperphosphorylation of RyR2 (Ser2808) in failing cardiomyocytes but had no effect on levels of phosphorylated PLB (Ser16 and Thr17). Low-dose landiolol significantly inhibited the alternans of CaT and CS under a fixed pacing rate (0.5 Hz) in failing cardiomyocytes. **Conclusion:** A low-dose β1-blocker in combination with milrinone improved cardiac function in failing cardiomyocytes, apparently by inhibiting the phosphorylation of RyR2, not PLB, and subsequent diastolic Ca^2+^ leak.

**INTRODUCTION**

**Background**

**3 a. Include sufficient scientific background (including relevant references to previous work) to understand the motivation and context for the study, and explain the experimental approach and rationale.**

**b. Explain how and why the animal species and model being used can address the scientific objectives and, where appropriate, the study’s relevance to human biology.**

The molecular mechanism underlying how low-dose β1-blocker combined with milrinone affects intracellular Ca^2+^ handling in heart failure remains unclear. One putative mechanism is through slowing HR, which decreases myocardial oxygen demand and improves diastolic filling [Circ J 2012;76:1646-1653.]. From several reports [Circulation 1956;14:1099-1103. Circulation 1992;85:1743-1750. Basic Res Cardiol 1996;91 Suppl 2:17-22. Am J Physiol 1991;261:H1746-1755. Pharmacol Ther 2008;119:340-354.], moreover, another contributing mechanism might be correction of aberrant intracellular Ca^2+^ handling. To demonstrate this hypothesis, we used cardiomyocytes isolated from dog normal hearts and failing hearts.

**Objectives**

**4 Clearly describe the primary and any secondary objectives of the study, or specific hypotheses being tested.**

To investigate the cardioprotective mechanism of a low-dose β1-blocker in intact failing canine cardiomyocytes to clarify the acute effect of low-dose β1-blocker on Ca^2+^ handling at a steady pacing rate of 0.5 Hz.

**METHODS**

**Ethical statement**

**5 Indicate the nature of the ethical review permissions, relevant licenses (e.g. Animal [Scientific Procedures] Act 1986), and national or institutional guidelines for the care and use of animals, that cover the research.**

This study conforms to the Guide for the Care and Use of Laboratory Animals published by the US National Institutes of Health (NIH Publication No. 85-23, revised 1996). All animal protocols were approved by the Yamaguchi University School of Medicine Animal Experiment Committee (institutional permission # 23-027).

**Study design**

**6 For each experiment, give brief details of the study design, including:**

**a. The number of experimental and control groups.**

**b. Any steps taken to minimize the effects of subjective bias when allocating animals to treatment (e.g., randomization procedure) and when assessing results (e.g., if done, describe who was blinded and when).**

**c. The experimental unit (e.g. a single animal, group, or cage of animals).**

**A time-line diagram or flow chart can be useful to illustrate how complex study designs were carried out.**

a-c. In 6 adult beagle dogs (10–13 kg), heart failure was induced by4 weeks rapid right ventricular pacing at 250 bpm. Six non-sham operated dogs were used as controls. Before sacrificing non-sham operated controls and 4weeks-pacing dogs, we measured heart rate, blood pressure, and indices of cardiac function by echocardiography in order to confirm that 4-weeks pacing induced heart failure (HF) under conscious condition.

**Experimental procedures**

**7 For each experiment and each experimental group, including controls, provide precise details of all procedures carried out. For example:**

**a. How (e.g., drug formulation and dose, site and route of administration, anaesthesia and analgesia used [including monitoring], surgical procedure, method of euthanasia). Provide details of any specialist equipment used, including supplier(s).**

**b. When (e.g., time of day).**

**c. Where (e.g., home cage, laboratory, water maze).**

**d. Why (e.g., rationale for choice of specific anaesthetic, route of administration, drug dose used).**

At the end of the study, dogs were euthanized with an isoflurane and intravenous injection of sodium pentobarbital and ventilated mechanically, followed by rapid removal of heart. Hearts were rapidly excised via thoracotomy. These procedures were performed at an animal operation room of Science Research Center at Yamaguchi University.

**Experimental animals**

**8 a. Provide details of the animals used, including species, strain, sex, developmental stage (e.g., mean or median age plus age range), and weight (e.g., mean or median weight plus weight range).**

**b. Provide further relevant information such as the source of animals, international strain nomenclature, genetic modification status (e.g. knock-out or transgenic), genotype, health/immune status, drug- or test naıve, previous procedures, etc.**

a. Dogs used in the present study, were all female beagle dogs (10-13 kg in body weight, 3-4 years old).

b. Dogs were obtained from KITAYAMA LABES CO., LTD (Japan).

**Housing and husbandry**

**9 Provide details of:**

**a. Housing (e.g., type of facility, e.g., specific pathogen free (SPF); type of cage or housing; bedding material; number of cage companions; tank shape and material etc. for fish).**

**b. Husbandry conditions (e.g., breeding program, light/dark cycle, temperature, quality of water etc. for fish, type of food, access to food and water, environmental enrichment).**

**c. Welfare-related assessments and interventions that were carried out before, during, or after the experiment.**

1. Housing: A large separate gage (D90xW85xH80) were given to each dog (number of gage was 12)
2. Husbandry condition: light /dark cycle (12hrs/12hrs) 7am-7pm; temperature 70^o^F ± 2 ^o^F; Food; food for experimental animals (TC-2, Oriental Yeast Co., LTD., Japan) was given every day. Water; drinking water.
3. Health check was performed by stuffs every day before and after pacemaker implantation in both operated group and non-sham operated group. If necessary, animal doctors saw dogs and treated them.

**Sample size**

**10 a. Specify the total number of animals used in each experiment and the number of animals in each experimental group.**

**b. Explain how the number of animals was decided. Provide details of any sample size calculation used.**

**c. Indicate the number of independent replications of each experiment, if relevant.**

Total number of dogs used in the present study was 12. Number of heart failure dogs (4 weeks pacing dog) was 6, while number of controls (non-sham operated controls was 6. There was significant difference in hemodynamic data (LVDD, LVDS, LVEF) between heart failure groups and controls (Please see Table 1).

**Allocating animals to experimental groups**

**11 a. Give full details of how animals were allocated to experimental groups, including randomization or matching if done.**

**b. Describe the order in which the animals in the different experimental groups were treated and assessed.**

a. N/A

b. The isolation of cardiomyocytes from control dogs and heart failure dogs was performed at same condition, and then Ca2+ transient and cell shortening, Ca2+ spark assay, and Western blot assay were also performed at the same condition.

**Experimental outcomes**

**12 Clearly define the primary and secondary experimental outcomes assessed (e.g., cell death, molecular markers, behavioural changes).**

In the present study, the primary experimental outcomes is to evaluate the effect of milrinone(+/-), landiolol(+/-) on Ca2+ transient and cell shortening, Ca2+ spark and phosphorylation levels of RyR and PLB in normal and failing cardiomyocytes.

**Statistical methods**

**13 a. Provide details of the statistical methods used for each analysis.**

**b. Specify the unit of analysis for each dataset (e.g. single animal, group of animals, single neuron).**

**c. Describe any methods used to assess whether the data met the assumptions of the statistical approach.**

The chi-squared test was used to compare prevalence or frequencies. The significance of differences between 2 groups was determined by post-hoc tests with Least Significant Difference algorithms following repeated-measures analysis of variance to evaluate the dose-dependence of landiolol on cell shortening in isolated cardiomyocytes. Comparisons across milrinone(+/-), landiolol(+/-), and heart failure(+/-) were independently verified with multivariate analysis of variance in experimental studies. Kruskal Wallis ANOVA was used to evaluate the antioxidative effect of landiolol on intact cardiomyocytes. All analyses were performed with SPSS 18.0 software (SPSS Inc., Chicago, Illinois). *P* values less than 0.05 were considered statistically significant.

**RESULTS**

**Baseline data**

**14 For each experimental group, report relevant characteristics and health status of animals (e.g., weight, microbiological status, and drug- or test-naıve) before treatment or testing (this information can often be tabulated).**

All animals analyzed were in good health.

**Numbers analyzed**

**15 a. Report the number of animals in each group included in each analysis. Report absolute numbers (e.g. 10/20, not 50%).**

**b. If any animals or data were not included in the analysis, explain why.**

Please refer to #6.

1. All animals in each group included in each analysis (100%).
2. N/A

**Outcomes and estimation**

**16 Report the results for each analysis carried out, with a measure of precision (e.g., standard error or confidence interval).**

Figure 1: N/A

Figure 2: The bars indicate the mean (SE).

Figure 3: N/A

Figure 4: The bars indicate the mean (SE).

Figure 5: The corresponding bar graphs, with bars indicating the mean (SE).

Figure 6: Each datum point represents the mean ± SE.

Table 1: Each datum point represents the mean ± SD.

**Adverse events**

**17 a. Give details of all important adverse events in each experimental group.**

**b. Describe any modifications to the experimental protocols made to reduce adverse events.**

There were no adverse events.

**DISCUSSION**

**Interpretation/scientific implications**

**18 a. Interpret the results, taking into account the study objectives and hypotheses, current theory, and other relevant studies in the literature.**

**b. Comment on the study limitations including any potential sources of bias, any limitations of the animal model, and the imprecision associated with the results.**

**c. Describe any implications of your experimental methods or findings for the replacement, refinement, or reduction (the 3Rs) of the use of animals in research.**

a. On the basis of our results, we propose the following model for the molecular basis of low-dose β-blocker treatment of ADHF (Please see Figure 7). First, in the baseline condition, enhanced phosphorylation of RyR2 Ser2808 induces Ca^2+^ leakage from SR, which causes intracellular Ca^2+^ overload and decreases [Ca^2+^]_SR_. Second, a low-dose β1-blocker selectively suppresses RyR2 Ser2808 hyperphosphorylation to inhibit Ca^2+^ leakage from SR but leave Ca^2+^ uptake through the sarco/endoplasmic reticulum Ca^2+^-ATPase unchanged. Third, monotherapy with milrinone selectively increases phosphorylation of PLB Ser16 and Thr17, but not to the extent of RyR2 Ser2808. Additionally, Ca^2+^ leakage from SR increases proportionally to increasing Ca^2+^ uptake. Eventually, the peak Ca^2+^ transient is slightly elevated. Fourth, combination therapy with milrinone and a low-dose β-blocker increases phosphorylation of PLB Ser16 and Thr17 and suppresses that of RyR2 Ser2808. These drugs also increase Ca^2+^ uptake and decrease Ca^2+^ leakage, which increases [Ca^2+^]_SR_ and the peak Ca^2+^ transient.

b. Inhibition of milrinone-induced diastolic Ca^2+^ leakage from the failing SR has been suggested to arise in part from selective inhibition of phosphorylated RyR2 (Ser 2808), the target amino acid of cAMP-dependent PKA. In the present study, however, we did not directly examine the effect of low-dose landiolol on phosphorylation of RyR2 (Thr 2814), the target amino acid of Ca^2+^/calmodulin-dependent protein kinase II (CaMK II). Recently, several reports indicated that CaMK II, rather than PKA, plays a critical role in diastolic Ca^2+^ leak through RyR2. Therefore, the mechanism by which low-dose landiolol suppressed milrinone-induced diastolic Ca^2+^ leak may also involve inhibition of RyR2 (Thr 2814) phosphorylation.

The phosphorylation level for PLB-Ser16 (PKA phosphorylated site) is much larger than PLB-Thr17 (CaMKII phosphorylated site) after addition of milrinone, which may suggest that milrinone affects Ca^2+^ handling through PKA phosphorylated site. Xiao B et al. reported that RyR2-Ser2030 site was the major phosphorylation site in RyR2 responding to PKA activation upon β adrenergic stimulation in normal and failing rat hearts. In the present study, however, we did not investigate the effect of milrinone and/or landiolol on the phosphorylation level of RyR2-Ser2030 in dog cardiomyocytes. Therefore, the mechanism by which low-dose landiolol suppressed Ca^2+^ leakage through RyR2 may be due to the inhibition of phosphorylation of RyR2-Ser2030 as well as the inhibition of phosphorylation of RyR2-Ser2808. Further research is needed to clarify these possibilities.

c. N/A

**Generalisability/translation**

**19 Comment on whether, and how, the findings of this study are likely to translate to other species or systems, including any relevance to human biology.**

The response of β1 blocker to β1 adrenergic receptor and response of intracellular Ca2+ handling to cAMP (milrinone) of dog heart are more similar to the responses of human heart than the responses to other small animals. Therefore, we used dog heart failure model. The proposed mechanism about low dose β1 blocker in the present study may be same as human heart failure.

**Funding**

**20 List all funding sources (including grant number) and the role of the funder(s) in the study.**

This work was funded by grants-in-aid for scientific research from the Ministry of Education in Japan (Grant No. 23592256 to SK and Grant No. 23390215 to MY), grant from Takeda Science Foundation in Japan to SK, and grant from SENSHIN Medical Research Foundation to SK. The funders had no role in study design, data collection and analysis, decision to publish, or preparation of the manuscript.
